# Distinguishing cubic and hexagonal phases within InGaN/GaN microstructures using electron energy loss spectroscopy

**DOI:** 10.1111/jmi.12285

**Published:** 2015-09-14

**Authors:** IJ GRIFFITHS, D CHERNS, S. ALBERT, A. BENGOECHEA‐ENCABO, M. ANGEL SANCHEZ, E. CALLEJA, T. SCHIMPKE, M. STRASSBURG

**Affiliations:** ^1^ School of Physics, H. H. Wills Physics LaboratoryUniversity of BristolBristolBS8 1TLUnited Kingdom; ^2^ ETSIT‐ISOMUniversidad Politécnica de Madrid, 28040 MadridSpain; ^3^ Osram Opto Semiconductors GmbH, Leibnizstrasse 493055RegensburgGermany

**Keywords:** EELS, InGaN micro‐structures, light emitting diodes, STEM

## Abstract

3D InGaN/GaN microstructures grown by metal organic vapor phase epitaxy (MOVPE) and molecular beam epitaxy (MBE) have been extensively studied using a range of electron microscopy techniques. The growth of material by MBE has led to the growth of cubic GaN material. The changes in these crystal phases has been investigated by Electron Energy Loss Spectroscopy, where the variations in the fine structure of the N K‐edge shows a clear difference allowing the mapping of the phases to take place. GaN layers grown for light emitting devices sometimes have cubic inclusions in the normally hexagonal wurtzite structures, which can influence the device electronic properties. Differences in the fine structure of the N K‐edge between cubic and hexagonal material in electron energy loss spectra are used to map cubic and hexagonal regions in a GaN/InGaN microcolumnar device. The method of mapping is explained, and the factors limiting spatial resolution are discussed.

## Introduction

**Figure 1:**
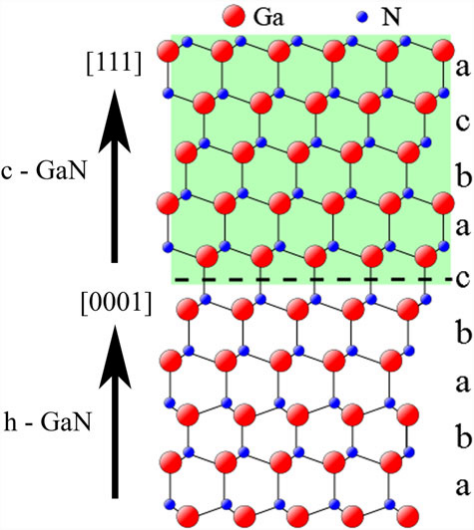
Schematic diagram showing the atomic arrangement between h‐GaN and c‐GaN.

The growth of 3D InGaN/GaN microstructures is of interest for the development of high efficiency light emitting diodes (LEDs) for solid state lighting, promising lower defect densities, lower carrier densities and reduced or eliminated piezoelectric fields, compared to conventional (0001)‐oriented thin films (Hong *et al*., 2011; Waag *et al*., 2011). However, the growth conditions employed in 3‐D growth can sometimes lead to a transformation from the expected hexagonal wurtzite structure to a cubic zinc blende structure (Novikov et al., 2011; Webster et al., 2014). This is significant for devices for several reasons, notably through differences in band structure between the hexagonal and cubic GaN, which for example, have bandgaps of 3.44 and 3.24 eV respectively. Such differences can be expected to lead to significant changes in carrier migration, trapping and recombination (Okumura et al., 1994; As & Mietze, 2013). The primary form for GaN crystal growth is the hexagonal phase, with lattice parameters of *a* = 0.3189 nm and *c* = 0.5185 nm. The cubic form has a lattice parameter of *a* = 0.450 nm, the cubic and hexagonal forms being related, most simply by a change of stacking sequence on (111) planes (Fig. 1).

**Figure 2:**
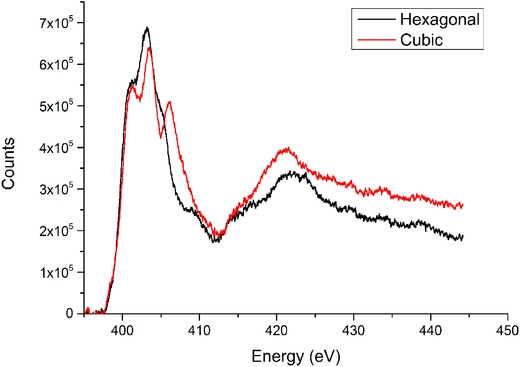
EELS spectra showing the N K‐edge taken from c‐GaN and h‐GaN.

Although the chemical compositions of c‐GaN and h‐GaN are identical, the differences in bonding structure leads to differences in the fine structure of the electron energy loss Spectroscopy (EELS) spectra for the atoms within the material. These differences are seen in both the Ga and N spectra. The most apparent differences are seen in the fine structure of the the N K‐edge, seen at 401 eV. Previous studies (Lazar et al., 2003) of the changes in the N K‐edge have shown a clear difference in the spectra between 405 and 408 eV (Fig. 2).

In this paper we examine a core‐shell structure exhibiting both hexagonal and cubic phases of GaN, and utilize the fine structure of the N K‐edge in electron energy loss spectroscopy (EELS) to map the different phases present. This work has been performed using a combination of transmission electron microscopy and scanning transmission electron microscopy.

## Experiment

**Figure 3:**
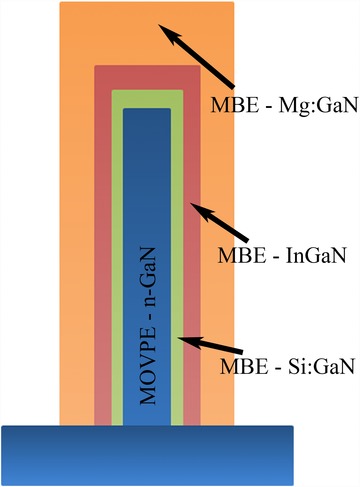
Diagram showing the structure of the 3D InGaN/GaN microstructures studied.

The InGaN/GaN microstructures have been grown on patterned c‐plane GaN‐on‐sapphire substrates using two growth techniques. The first stage in the growth has been performed using continuous flow metal organic vapour phase epitaxy (MOVPE), producing defect free n‐doped GaN microrods with a high aspect ratio. The second growth stage has been performed using molecular beam epitaxy (MBE) to produce multiple InGaN layers and subsequent p‐doped GaN (Fig. 3). The growth of the p‐GaN has been performed at a lower temperature, 625 °C, than that of the initial MBE n‐GaN, 925 °C, to prevent desorption of the indium from the InGaN layers.

The samples have been prepared for analysis using a dual‐beam focused ion beam (FIB) to ensure an accurate sample location. The initial FIB preparation has been performed with an operating voltage of 30kV. To reduce the surface damage created by the Ga ion etch process, a lower energy (8 kV) polish has been used.

The samples have been studied using a JEOL 2010 TEM, and a JEOL ARM 200F TEM/STEM both operating at 200kV. The EELS characterisation has been performed using a Gatan GIF Quantum 965 ER, utilising the Dual EELS capability to correct for drift in the core‐loss signal. Analysis of the spectra has been performed in Digital Micrograph, using the multiple linear least square (MLLS) fitting routine to determine a fit to assigned spectra.

## Results

**Figure 4:**
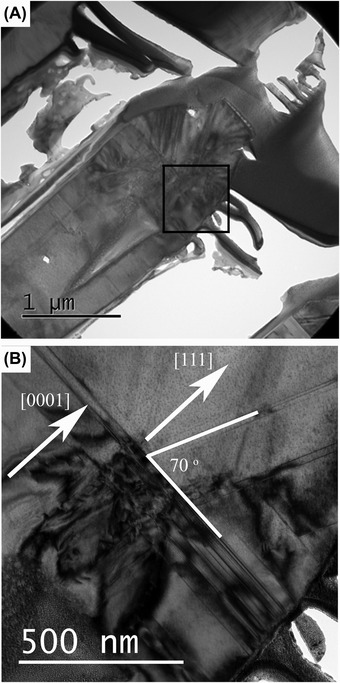
(A) A low‐magnification TEM image showing the overall structure. (B) The interface between the h‐GaN and the c‐GaN.

Initial studies of the samples by TEM and diffraction show microstructures with a high aspect ratio defect free core, and a highly defective region towards the top of the structure containing a high density of basal plane stacking faults. As seen in Figure 4, the top of the structure shows cubic morphology with stacking faults at 70^o^ to the c‐plane within the h‐GaN. The cubic structure at the top region of the microstructure has been confirmed by selected area electron diffraction, with a clear (111) orientation. The basal plane stacking faults are clearly visible in the hexagonal region extending across the structure, along with stacking faults visible in the cubic material.

**Figure 5:**
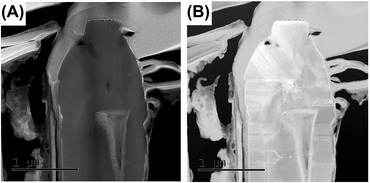
(A) HAADF STEM image revealing the Z‐contrast in the microstructure. (B) MAADF image showing diffraction contrast revealing the defects.

Analysis of the sample by high angle annular dark field (HAADF) has been used to view the Z‐contrast within the structure. A HAADF image is shown in Figure 5A, where it is possible to determine the location of the InGaN active region around the MOVPE n‐GaN core. When the sample is viewed under medium angle annular dark field (MAADF) conditions (Fig. 5B), it is possible to reveal some of the defects seen under conventional TEM conditions. The inner collection angles for the HAADF and MAADF detectors are 76.9 and 31.3 mrad, respectively. Comparing the HAADF and MAADF images it is possible to determine the initial basal plane stacking faults (BSFs) nucleate after the growth of the InGaN region, suggesting the change in conditions after the growth of the active region encourages the nucleation of defects and ultimately cubic material.

In order to map the cubic regions of the microstructures, an area known to contain both crystal forms was mapped with a large pixel size to ensure a high signal to noise ratio. To create a fuller representation of the pixels studied, subpixel scanning was applied during the acquisition of the EELS spectra. During this process, the STEM beam is rastered across the entire pixel dimensions instead of moving from one pixel to the next. Due to the large area covered in the scan, Dual EELS was used to record the zero‐loss peak simultaneous to the core‐loss signal. This technique is important as it allows for drift correction of the core‐loss signal. The acquisition of the zero‐loss peak has also allowed the sample thickness to be determined and used to normalise the acquired maps as a function of thickness. For the selected area, the thickness varied in the range 80–120 nm.

**Figure 6:**
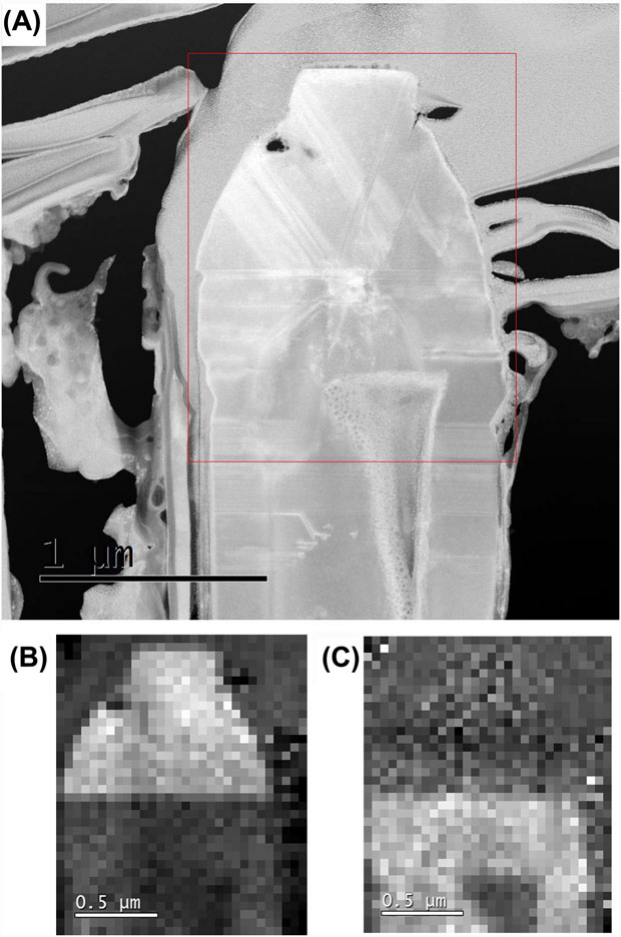
(A) MAADF STEM image showing the area mapped. (B) A map revealing the regions of c‐GaN. (C) The h‐GaN map showing the main microstructure.

In order to create a crystal phase map for the top of the microstructure, a large pixel size of 50 nm was used, with a scan area of 1.5 μm × 2 μm. A MAADF image showing the scan area is shown in Figure 6A, where the presence of planar defects is clearly visible. The EELS spectra recorded were corrected for drift and the core‐loss was extracted from the background to reveal the N K‐edge (Fig. 2). By applying MLLS fitting, over the range of 404–408 eV, to the spectral map it is possible to determine the crystal phases present. The extracted maps are shown in Figures 6B and C. These maps have been corrected for thickness variations, and to exclude the signal from non‐GaN material. The maps show the presence of cubic GaN in the top region of the microstructure, with the remaining material being hexagonal GaN. The area of the microstructure within the h‐GaN region with little fitting is due to heavy structural etch damage as a result of sample preparation. This can be seen in both the HAADF and MAADF images.

**Figure 7:**
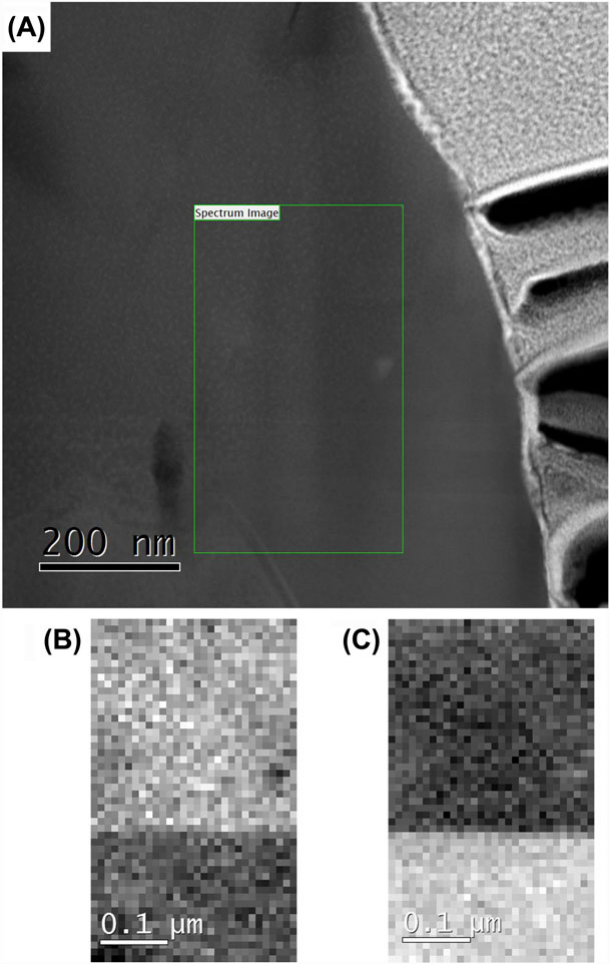
(A) HAADF image showing the location of the map. (B) The c‐GaN region. (C) The h‐GaN region showing a clear interface with the c‐GaN.

To increase the resolution of the phase maps obtained, the pixel size of the spectrum images was reduced to 10 nm pixels. The ultimate resolution of the maps is limited by the signal to noise ratio, along with the ratio of the peaks within the fine structure. A map was performed with a size of 250 nm × 500 nm, with the scan area shown in Figure 7A on a HAADF image. In the lower left corner of the scan area, the InGaN active region can be seen by the change in contrast associated with InGaN. Due to the lack of diffraction contrast present in HAADF images, the interface between the h‐GaN and the c‐GaN is hard to determine. The spectra acquired for this area were processed in the same way as the previous data, allowing clear maps of the c‐GaN region and the h‐GaN region to be created (Figs. 7B and C).

## Discussion

InGaN/GaN 3D microstructures grown by MOVPE and MBE have been studied via a range of electron microscopy techniques to characterise the crystalline nature of the material. The structures have been studied using electron energy loss spectroscopy to reveal differences in the N K‐edge present in the c‐GaN and h‐GaN spectra. These differences have revealed the presence of cubic GaN at the top of the structures in the region immediately after the growth of the InGaN active region. This growth is believed to occur as a result of the lower growth temperature required to prevent changes to the In concentrations within the InGaN layers. The studies have revealed that with a pixel size of 10 nm it is possible to clearly distinguish between the different phases present. Further work in this material will aim to reduce the pixel size to sub nanometre resolution allowing the properties of the material containing a high density of basal plane stacking faults to be studied.
